# Effectiveness of Sodium-Glucose Cotransporter-2 Inhibitors vs. Dipeptidyl Peptidase-4 Inhibitors in Frail People With Diabetes Who Were Recently Hospitalized

**DOI:** 10.3389/fphar.2022.886834

**Published:** 2022-07-12

**Authors:** Stephen J Wood, J Simon Bell, Dianna J Magliano, Jonathan E Shaw, Matteo Cesari, Jenni Ilomaki

**Affiliations:** ^1^ Centre for Medicine Use and Safety, Faculty of Pharmacy and Pharmaceutical Sciences, Monash University, Melbourne, VIC, Australia; ^2^ Department of Epidemiology and Preventive Medicine, Monash University, Melbourne, VIC, Australia; ^3^ National Health and Medical Research Council Centre of Research Excellence in Frailty and Healthy Ageing, Adelaide, SA, Australia; ^4^ Baker Heart and Diabetes Institute, Melbourne, VIC, Australia; ^5^ IRCCS Istituti Clinici Scientifici Maugeri, University of Milan, Milan, Italy

**Keywords:** SGLT-2i, frailty, type 2 diabetes, MACE, heart failure

## Abstract

**Introduction:** Sodium-glucose cotransporter-2 inhibitors (SGLT-2Is) reduce heart failure (HF) hospitalizations and major adverse cardiovascular events (MACE) in general type 2 diabetes populations. The objective of this study was to determine whether SGLT-2Is vs. dipeptidyl peptidase-4 inhibitors (DPP-4Is) are associated with reductions in MACE, HF hospitalizations and mortality in frail people with type 2 diabetes.

**Methods:** We conducted a cohort study of all patients aged ≥30 years with type 2 diabetes discharged from a hospital in Victoria, Australia between January 2014 and March 2018 who received SGLT-2Is or DPP-4Is within 60 days of discharge. Follow-up commenced 60 days after initial discharge, and MACE, HF hospitalization and mortality were recorded. Cox proportional hazards regression with competing risks and stabilized inverse probability of treatment weights (IPTWs), was used to generate subdistribution hazard ratios (sHRs) with 95% confidence intervals (CIs). Analyses were stratified into frailty quartiles according to Hospital Frailty Risk Scores (HFRS).

**Results:** Of the 32,043 patients, (41.9% female and 5.9% ≥80 years) in the cohort, 5,152 (16.1%) received SGLT-2Is. Overall, SGLT-2I versus DPP-4I recipients had lower rates of MACE (sHR 0.51; 95% CI 0.46–0.56), HF hospitalization (sHR 0.42; 95% CI 0.36–0.49) and mortality (HR 0.38; 95% CI 0.33–0.43). People with HFRSs in the fourth quartile who received SGLT-2Is versus DPP-4Is also had reduced rates of MACE (sHR 0.37; 95% CI 0.29–0.46), HF hospitalization (sHR 0.43; 95% CI 0.33–0.56) and mortality (HR 0.32; 95% CI 0.25–0.41).

**Conclusion:** SGLT-2Is may be preferred to DPP-4Is for preventing MACE, HF hospitalizations and mortality in frail people with type 2 diabetes.

## Introduction

Randomized controlled trials (RCTs) demonstrate sodium-glucose cotransporter-2 inhibitors (SGLT-2Is) reduce hospitalizations for heart failure (HF) and mortality in general older populations with type 2 diabetes (Zinman et al., 2015; Perkovic et al., 2018; Filion et al., 2020). However, despite an estimated 32–48% prevalence of frailty in people with diabetes (Perkisas and Vandewoude, 2016), people who are frail are often excluded from RCTs. There is increasing interest in whether treatment benefits and risks in general older populations can be extrapolated to people who are frail (Onder et al., 2018). This is important because frailty is a medical condition closely related to diabetes and a risk factor for diabetes-related complications (Abdelhafiz et al., 2016).

Clinical practice guidelines recommend prescribing second-line therapies when metformin or sulfonylureas are not tolerated or are unsuccessful in controlling hyperglycemia (The Royal Australian College of General Practitioners (RACGP), 2016; Garber et al., 2020; National Institute for Health and Care Excellence (NICE), 2017), but clinicians treating frail older people with type 2 diabetes face challenges selecting appropriate second-line therapy. Systematic reviews have shown people who are frail have over 5-times higher odds of hospitalization and a 35% increased risk of mortality compared to non-frail individuals with diabetes (Ida et al., 2019). SGLT-2Is and dipeptidyl peptidase-4 inhibitors (DPP-4Is) do not cause hypoglycemia, are administered orally (The Royal Australian College of General Practitioners (RACGP), 2016), and may be preferred over sulfonylureas and insulin in people at high risk of hypoglycemia such as those who are frail (Ibrahim et al., 2020). We have previously demonstrated that people who are frail are less likely to be prescribed insulin at hospital discharge than those who are non-frail (Wood et al., 2021). It remains unclear whether SGLT-2Is or DPP-4Is have the same benefits and risks in frail people with type 2 diabetes compared to non-frail people with type 2 diabetes.

In general populations of people with type 2 diabetes, meta-analyses have shown that DPP-4Is do not reduce the risk of major adverse cardiovascular events (MACE) compared to placebo (Kaneko and Narukawa, 2016). The Saxagliptin Assessment of Vascular Outcomes Recorded in Patients with Diabetes Mellitus (SAVOR)–Thrombolysis in Myocardial Infarction 53 (TIMI-53) trial concluded that the DPP-4I saxagliptin did not reduce ischemic events but increased HF hospitalizations by 27% (Scirica et al., 2013). Overall, however, there is no evidence that DPP-4Is increase the risk of MACE or HF (Karagiannis et al., 2016). In contrast, some cardiovascular benefits of SGLT-2Is are well established (Zinman et al., 2015; Perkovic et al., 2018; Wiviott et al., 2018). The Empagliflozin, Cardiovascular Outcomes, and Mortality in type 2 Diabetes (EMPA-REG OUTCOMES) trial (Zinman et al., 2015) demonstrated 38% relative risk reduction in cardiovascular death, 32% reduction in all-cause mortality, and 35% reduction in HF hospitalizations. However, it was not shown to significantly affect rates of myocardial infarction (MI) or stroke (Zinman et al., 2015). A network meta-analysis by Fei et al. found that SGLT-2Is were associated with 17% lower odds of both cardiovascular and all-cause death compared to DPP-4Is (Fei et al., 2019).

To our knowledge, no studies to date have investigated whether frailty modifies the effect of SGLT-2Is on MACE, HF hospitalization, and mortality in people with type 2 diabetes. However, considering the advantages of SGLT-2Is reducing HF hospitalizations in people with type 2 diabetes, we hypothesized that benefits would be evident in this vulnerable population. The objective of this study was to determine whether SGLT-2Is, compared to DPP-4Is, prevent MACE, HF hospitalizations and mortality in frail people with type 2 diabetes.

## Research Design and Methods

### Data Source, Study Design, and Study Population

We utilized data from the Victorian Admitted Episodes Dataset (VAED). This dataset contains demographic and administrative information, and diagnostic and procedural codes for all episodes of care across Victorian public and private hospitals, rehabilitation centres, extended care facilities, and day procedure centres (State Government of Victoria, 2019). Victoria is Australia’s second most populous state with a population of 6.7 million. The VAED includes hospitalization records from 2006 until June 2018 for all individuals hospitalized with a recorded type 2 diabetes diagnosis at any time during this period. VAED data were linked to data on medication dispensing through Australia’s Pharmaceutical Benefits Scheme (PBS). The PBS subsidizes the cost of medications dispensed through community pharmacies and at hospital discharge for all Australian citizens, residents and visitors from countries with reciprocal health coverage. Data were also linked to the National Death Index for dates and causes of death. Data linkage was performed by the Australian Institute of Health and Welfare (AIHW). Ethics approval was acquired from AIHW Ethics Committee (EO2018-4-468) and Monash University Human Research Ethics Committee (14339).

**FIGURE 1:**
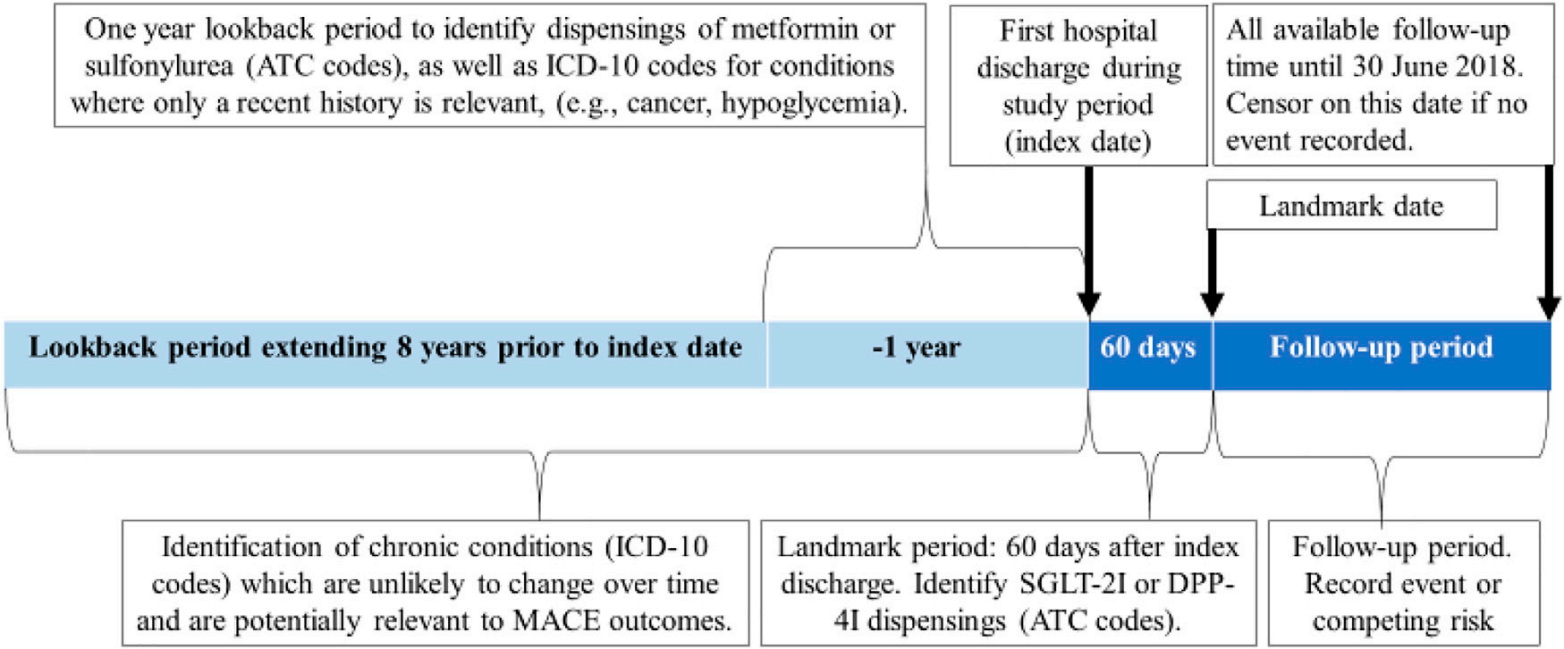
An illustration depicting the study design.

**FIGURE 2:**
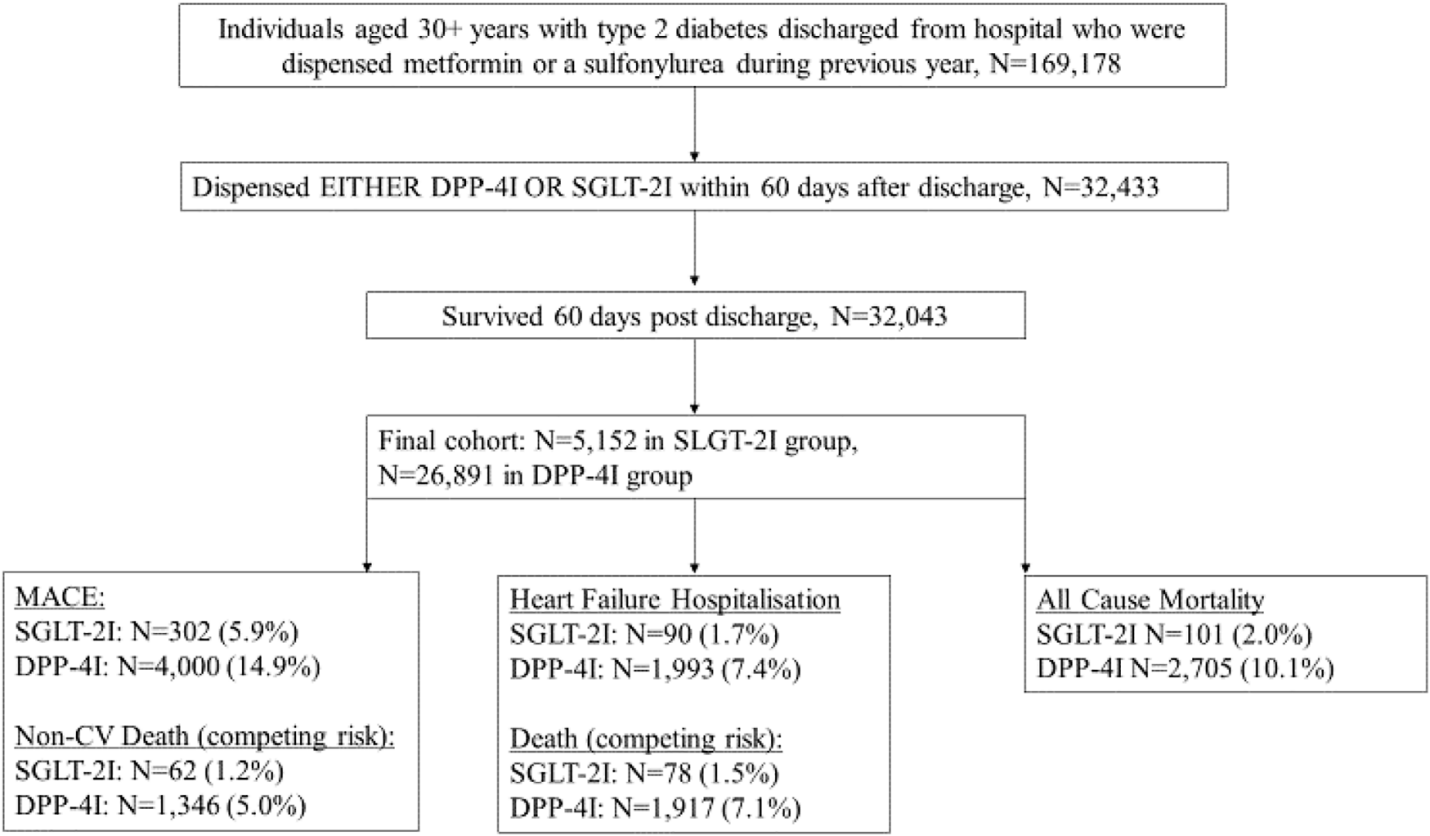
A flowchart indicating how cohort was obtained and numbers of outcomes.

We conducted a cohort study on the effects of SGLT-2Is compared to DPP-4Is in the prevention of MACE, HF hospitalization, and mortality during the first year after hospital discharge. The cohort comprised people aged ≥30 years with type 2 diabetes who were discharged from hospital between January 2014 and March 2018. We only included people who used metformin or sulfonylurea as their first-line treatment because Australian PBS regulations stipulate that SGLT-2Is or DPP-4Is can only be subsidized for people who have trialed one of these first line therapies without meeting glycemic targets. Additionally, this approach reduced the risk of confounding by disease severity. The use of metformin and sulfonylureas was captured from PBS dispensing at or 365 days prior to the initial discharge date (index date) (Figure 1). Exposure to SGLT-2Is and DPP-4Is was assessed during a landmark period of 60 days after the index date for each patient (Figure 1). The landmark period methodology was chosen as it is the preferred method to minimize immortal-time bias (Mi et al., 2016) and it permits the exclusion of people who die soon after their initial hospitalization. This method ensures that all individuals survived an equal minimum length of time after the index hospitalization to avoid differential immortal times between the groups. We selected a 60-days period in order to capture the majority of SGLT-2I or DPP-4I users. This is because in Australia SGLT-2Is and DPP-4Is are usually dispensed in quantities that correspond to 28–30 days of treatment, but some people miss doses or have additional supplies from a previous dispensing. The follow-up commenced after the 60-days landmark period. Patients who died or received both an SGLT-2I and a DPP-4I during the landmark period were excluded from the study. MACE outcomes during the landmark period were not recorded as outcomes, because there may have been insufficient time for the medications to exert an effect by this point (Figure 2).

### Measures and Definitions

Anatomical Therapeutic Chemical (ATC) codes were used to identify SGLT-2I (canagliflozin, empagliflozin, dapagliflozin and ertugliflozin) and DPP-4I (sitagliptin, vildagliptin, saxagliptin, linagliptin and alogliptin) dispensings during the landmark period and to identify other relevant medications dispensed during the year before the index date (Supplementary Appendix S1A). The latter included a range of cardiovascular medication classes as well as antipsychotics, owing to their effects on glucose levels. ATC codes were also used to identify dispensings of DPP-4Is during the year prior to index discharge. The International Classification of Diseases-10 (ICD-10) codes were used to identify diagnoses for type 2 diabetes (E11) as well as chronic diseases including cardiovascular conditions, dementia, and diabetic complications (Supplementary Appendix S1B) (Dugan and Shubrook, 2017). These were identified using the previous 8 years of hospital admissions data, prior to the index date of each patient. Acute conditions such as severe hypoglycemia and conditions which can change substantially over time, such as cancer, were identified from the hospital admissions data using a 1-year lookback period (Supplementary Appendix S1B).

MACE has various definitions (Fei et al., 2019; Filion et al., 2020), but we used the definition which captured the broadest possible range of cardiac outcomes. MACE was identified using ICD-10 codes (MI, HF hospitalization, and stroke) and ICD-10 procedure codes [Percutaneous Coronary Interventions (PCIs) with stents and Coronary Artery Bypass Grafts (CABGs) and revascularization; Supplementary Appendix S1C]. If a patient died during follow-up without hospitalization for MACE, and the ICD-10 code identifying their primary cause of death was indicative of MACE, then an event was recorded. If the primary cause of death was unrelated to MACE, then the person was deemed to have experienced a competing risk on the death date. In the HF hospitalization analysis, the ICD-10 code “I50” was considered an event, whereas all-cause death was recorded as a competing risk for those without hospital admission for HF. In the all-cause mortality analyses, the outcome was death due to any cause during the follow-up period. In all analyses, patients who did not experience an event or did not die were censored on 30^th^ June 2018.

To identify people who are frail, we utilized the validated Hospital Frailty Risk Score (HFRS). The HFRS quantifies frailty based on the sum of weighted scores identified from International Classification of Diseases (ICD-10) codes (Supplementary Appendix S1D) (Gilbert et al., 2018). Gilbert et al. (2018) derived this score using 109 ICD-10 codes at least twice as prevalent in frail versus non-frail patients weighted according to how strongly they predict frailty (Gilbert et al., 2018). Codes used to derive the HFRS reflect conditions linked to frailty (for example, volume depletion, cognitive impairment, and falls) or conditions overrepresented in frail populations such as lung disease, heart conditions, and elective cataracts.

To account for diabetes severity, we used a modified version (Glasheen et al., 2017) of the Diabetes Complications Severity Index (DCSI). This version of the DCSI utilizes ICD-10 codes to produce a 14-level metric, which quantifies the effects of type 2 diabetes on seven different organ systems (Supplementary Appendix S1E). It has also been found to be significantly positively associated with the number of hospitalizations over 4 years, despite not requiring laboratory test results for its calculation (Chang et al., 2012).

### Statistical Analysis

We stratified the cohort into three categories based on HFRS. Those with a HFRS of 0 constituted over 50% of the cohort. We considered people with a HFRS in the third and fourth quartile as being frail. This ensured sufficient sample sizes within each stratum.

We used Standardized Mean Difference (SMD), produced using the stdiff function of SAS, to compare differences in baseline characteristics between the treatment and comparator group. SMD was calculated by taking the difference of sample means between the treatment and comparator groups for each covariate and dividing by the square root of the average sample variance of the treatment and comparator groups (Austin, 2009). SMDs >10% indicated suboptimal balance of the characteristic between groups. We utilized Cox Proportional Hazards Regression with Fine and Gray competing risks to estimate the effect between SGLT-2I use versus DPP-4I use against HF hospitalization and MACE. We accounted in all three models for clinical differences between people dispensed SGLT-2Is and those dispensed DPP-4Is using Stabilized Inverse Probability of Treatment Weights (IPTW). Stabilized IPTWs assigned to those given treatment were calculated by dividing the probability of being assigned to the treatment group divided by the conditional probability of being assigned to the treatment group, given other baseline characteristics. Similarly, the stabilized IPTW for those in the comparator group was calculated by dividing the probability of being in the comparator group by the conditional probability of being in the comparator group, given the specific set of baseline covariates (Thoemmes and Ong, 2015). Stabilized IPTWs were also used to estimate weighted SMDs (Table 1). We also repeated the analysis using an interaction term (treatment*HFRS) in the multivariate model. Subdistribution hazard ratios (sHR) were estimated for MACE and HF hospitalization and hazard ratios (HR) were estimated for all-cause mortality. All analyses were conducted using the statistical software package SAS version 9.4 (SAS Institute Inc., Cary, NC, United States).

**TABLE 1:** Baseline characteristics of patients hospitalised with type 2 diabetes with a history of metformin or sulfonylurea dispensings in the year prior to index discharge.

	Total	SGLT-2I	DPP-4I	Unweighted Standardized Difference	Weighted Standardized Difference
	N = 32,043	N = 5,152	N = 26,891	(%)	(%)
**Age, years, (n, %)**				−3.95	−5.44
30-59	12,425 (38.8)	2,895 (56.2)	9,530 (35.4)		
60-69	10,875 (33.9)	1,668 (32.4)	9,207 (34.2)		
70-79	6,857 (21.4)	536 (10.4)	6,321 (23.5)		
80+	1,886 (5.9)	53 (1.0)	1,833 (6.8)		
**Sex (n, %)**				5.01	0.72
Female	13,431 (41.9)	2,053 (39.8)	11,378 (42.3)		
**Index discharge year, (n, %)**				−3.95	−5.44
2014	9,330 (29.1)	232 (4.5)	9,098 (33.8)		
2015	7,455 (23.3)	794 (15.4)	6,661 (24.8)		
2016	6,946 (21.7)	1,562 (30.3)	5,384 (20.0)		
2017	6,783 (21.2)	2,037 (39.5)	4,746 (17.6)		
2018^a^	1,529 (4.8)	527 (10.2)	1,002 (3.7)		
**Hospital Frailty Risk Score, (n, %)**				−3.76	−0.65
0 (1st and 2nd quartile)	17,581 (54.9)	3,249 (63.1)	14,332 (53.3)		
0.1–1.8 (3rd quartile)	6,727 (21.0)	1,016 (19.7)	5,711 (21.2)		
>1.8 (4th quartile)	7,735 (24.1)	887 (17.2)	6,848 (25.5)		
**Diabetes Complications Severity Index (n, %)**				8.14	−9.34
0	26,036 (81.3)	4,228 (82.1)	21,808 (81.1)		
1	3,218 (10.0)	627 (12.2)	2,591 (9.6)		
≥2	2,789 (8.7)	297 (5.8)	2,492 (9.3)		
**Admitted to hospital with MACE** ^a^	2,443 (7.6)	330 (6.4)	2,113 (7.9)	−5.65	−5.75
**Medications used up to 1 year prior to discharge (n, %)**					
ACE inhibitors/ARB	24,302 (75.8)	3,903 (75.8)	20,399 (75.9)	−0.24	−11.03
Beta-blockers	9,485 (29.6)	1,349 (26.2)	8,136 (30.3)	−9.06	1.10
Calcium channel blockers	6,051 (18.9)	766 (14.9)	5,285 (19.7)	−12.69	1.29
Statin	25,372 (79.2)	4,127 (80.1)	21,245 (79.0)	2.73	−8.83
MRA	1,610 (5.0)	217 (4.2)	1,393 (5.2)	−4.58	−1.42
Digoxin	1,262 (3.9)	115 (2.2)	1,147 (4.3)	−11.49	−4.13
Diuretics (thiazide and loop)	5,174 (16.1)	480 (9.3)	4,694 (17.5)	−24.08	−6.31
Oral anticoagulant	1,673 (5.2)	120 (2.3)	1,553 (5.8)	−17.54	−9.96
Antiplatelet	7,019 (21.9)	770 (14.9)	6,249 (23.2)	−21.22	−3.80
Antipsychotics	1,525 (4.8)	221 (4.3)	1,304 (4.8)	−2.68	3.29
Prior use of DPP-4Is	25,727 (80.3)	818 (15.9)	24,909 (92.6)	−241.61	8.54
**Medical Conditions Prior to Index Discharge (n, %)**					
Unstable Angina	1,268 (4.0)	146 (2.8)	1,122 (4.2)	−7.29	−1.99
Angina pectoris	1,354 (4.2)	167 (3.2)	1,187 (4.4)	−6.12	1.76
Peripheral vascular disease	990 (3.1)	95 (1.8)	895 (3.3)	−9.36	−3.20
Myocardial infarction	841 (2.6)	132 (2.6)	709 (2.6)	−0.47	−10.82
Hypertension	9,200 (28.7)	871 (16.9)	8,329 (31.0)	−33.42	−0.51
Heart failure	2,120 (6.6)	155 (3.0)	1,965 (7.3)	−19.53	−1.17
Atrial fibrillation	2,678 (8.4)	232 (4.5)	2,446 (9.1)	−18.32	−2.67
Stroke	1,060 (3.3)	113 (2.2)	947 (3.5)	−7.98	3.51
Chronic Obstructive Pulmonary Disease	1,438 (4.5)	150 (2.9)	1,288 (4.8)	−9.77	−1.44
Cancer	2,252 (7.0)	248 (4.8)	2,004 (7.5)	-11.01	-6.52
Severe hypoglycaemia	103 (0.3)	14 (0.3)	89 (0.3)	-1.08	-5.78
Dialysis	98 (0.3)	<6^c^	Not reported^c^	-6.52	12.57
Chronic kidney disease	5,261 (16.4)	436 (8.5)	4,825 (17.9)	-28.28	4.76
Diabetic polyneuropathy	1,866 (5.8)	281 (5.5)	1,585 (5.9)	-1.90	8.53
Diabetic eye disease	5,473 (17.1)	697 (13.5)	4,776 (17.8)	-11.67	-8.42
Diabetic foot	1,527 (4.8)	183 (3.6)	1,344 (5.0)	-7.15	-7.38
Other diabetic complications	12,988 (40.5)	1,962 (38.1)	11,026 (41.0)	-5.98	3.38
Dementia	2,029 (6.3)	434 (8.4)	1,595 (5.9)	9.67	-3.37

a Only those with an index hospital discharge in the first quarter of 2018 were included.

b MACE, major adverse cardiovascular event includes myocardial infarction, heart failure, Percutaneous Coronary Intervention (PCI), Coronary Artery Bypass Graft (CABG), thrombolysis and stroke.

c Figures <6, or other figures enabling the calculation of these numbers, cannot be reported. SGLT-2I, Sodium Glucose Cotransporter-2 inhibitors; DPP-4I, Dipeptidyl Peptidase-4 inhibitors; ACE, angiotensin converting enzyme inhibitor; ARB, Angiotensin-2 receptor Blocker; MRA, mineralocorticoid receptor antagonist.

## Results

### Cohort Characteristics

In total there were 32,043 patients included in the cohort, with 5,152 (16.1%) dispensed SGLT-2Is and 26,891 dispensed DPP-4Is (Table 1). People receiving SGLT-2Is after hospital discharge were younger, with 56.2% being between 30 and 59 years (35.4% among people who were dispensed DPP-4I). The respective proportions of those in the SGLT-2I and DPP-4I groups aged 80 or over were 1.0 and 6.8%. Among those who were dispensed SGLT-2Is, 39.8% were women, while 42.3% of those dispensed DPP-4Is were women. Among SGLT-2I and DPP-4I recipients, 15.9 and 92.6% had received DPP-4Is during the year prior to index discharge. Conversely, of those identified as receiving DPP-4Is in the year prior to index date, 96.8% were in the DPP-4I cohort and 3.2% were in the SGLT-2I cohort (Supplementary Appendix S1F). MACE was the primary reason for hospitalization in 6.4% of the SGLT-2I and 7.9% of the DPP-4I cohorts.

People in frailty quartiles 1 and 2 all had HFRS scores of 0, collectively these individuals represented 54.9% of the cohort (Table 1). The proportion of people in the fourth frailty quartile was 17.2% among SGLT-2I recipients and 25.5% in DPP-4I recipients. DCSI scores ≥2 were found in 5.8% of SGLT-2I recipients and 9.3% of DPP-4I recipients. At baseline, people prescribed DPP-4Is, compared to SGLT-2Is, had a higher prevalence of hypertension, (31.0 versus 16.9%), HF, (7.3 versus 3.0%), atrial fibrillation (AF), (9.1 versus 4.5%) and chronic kidney disease (CKD), (17.9 versus 8.5%). However, dementia was more prevalent amongst those dispensed SGLT-2Is compared to DPP-4Is (8.4 versus 5.9%). Stabilized IPTW resulted in SMDs <10% for most variables except ACE/ARB (angiotensin converting enzyme inhibitors or angiotensin-2 receptor blockers), (−11.03%) prior MI (−10.82%), and dialysis (12.57%).

MACE or HF hospitalization occurred in 5.9 and 1.7% of the SGLT-2I group and 14.9 and 7.4% of the DPP-4I group, respectively, during the follow-up period. 2.0% of the SGLT-2I group and 10.1% of the DPP-4I group died.

Among the entire cohort, the rates of MACE were significantly lower in those receiving SGLT-2Is (sHR 0.51; 95% CI 0.46–0.56) compared to those receiving DPP-4Is. SGLT-2I recipients in the third (sHR 0.39; 95% CI 0.31–0.49) and fourth (sHR 0.37; 95% CI 0.29–0.46) frailty quartiles also had lower rates of MACE than DPP-4I recipients.

The HF hospitalization rate for the cohort was lower for those receiving SGLT-2Is, compared to DPP-4Is (sHR 0.42; 95% CI 0.36–0.49). HF hospitalization rates were also lower among SGLT-2I recipients, compared to DPP-4I recipients for those in the third (sHR 0.26; 95% CI 0.17–0.39) and fourth (sHR 0.43; 95% CI 0.33–0.56) frailty quartiles. All-cause mortality (HR 0.38; 95% CI 0.33–0.43) rates were reduced among the cohort as a whole for individuals dispensed SGLT-2Is compared to DPP-4Is, and this was also observed in the third (HR 0.21; 95% CI 0.15–0.30) and fourth (HR 0.32; 95% CI 0.25–0.41) frailty quartiles.

In the analysis which included an interaction term defined as the product of treatment group and the HFRS (Table 2), the magnitude and direction of the estimates were similar to the main analysis for MACE (sHR 0.43; 95% CI 0.38–0.49). However, the interaction term resulted in lower estimates in the rates of HF hospitalization (sHR 0.27; 95% CI 0.21–0.34), and all-cause mortality (HR 0.24; 95% CI 0.20–0.29), compared to the model without the interaction term.

**TABLE 2:** Rates of major adverse cardiovascular events, heart failure hospitalisation and all-cause mortality in patients with type 2 diabetes dispensed sodium glucose Cotransporter-2 inhibitors versus dipeptidyl Peptidase-4 inhibitors, stratified by frailty status.

Cohort	MACE	Heart Failure Hospitalization	All-Cause Mortality
	sHR; 95% CI^a^	sHR; 95% CI^b^	HR; 95% CI
All individuals with T2D ≥ 30 years, N = 32,043	0.51 (0.46–0.56)	0.42 (0.36–0.49)	0.38 (0.33–0.43)
All individuals with T2D ≥ 30 years. Including treatment*HFRS interaction. N = 32,043	0.43 (0.38–0.49)	0.27 (0.21–0.34)	0.24 (0.20–0.29)
HFRS = 0 N = 17,581	0.54 (0.47–0.62)	0.30 (0.23–0.39)	0.47 (0.38–0.59)
0 ≤ HFRS≤1.8 N = 6,727	0.39 (0.31–0.49)	0.26 (0.17–0.39)	0.21 (0.15–0.30)
HFRS> 1.8 N = 7,735	0.37 (0.29–0.46)	0.43 (0.33–0.56)	0.32 (0.25–0.41)

Cox Proportional Hazards Regression was used with estimates adjusted for variables in Table 1, using Stabilized Inverse Probability Weights (IPTW); HF, heart failure; HFRS, hospital frailty risk score; HR, hazard ratio; MACE, major adverse cardiovascular events; sHR, subdistribution hazard ratio.

a Competing risk of all non-MACE, mortality.

b Competing risk of all-cause mortality.

## Discussion

A key finding of our study was that SGLT-2Is were associated with similar reductions in rates of MACE in people who are frail and non-frail. We found that SGLT-2Is are associated with a 49% reduced rate of MACE compared to DPP-4Is in adults aged ≥30 years. People in the third and fourth frailty quartiles had approximately 60% lower rates of MACE when dispensed SGLT-2Is compared to DPP-4Is. These results were directionally consistent with the sensitivity analysis, which included a term to adjust for the effect modification of HFRS on treatment group. Our findings that SGLT-2Is are associated with reduced rates of MI or stroke were consistent with the multi-national CVD-REAL2 study, which showed reduced risk of MI or stroke by 12 and 15%, respectively, compared to DPP-4Is (Kohsaka et al., 2020). In the CVD-REAL2 study (Kosiborod et al., 2018), SGLT-2Is were found to reduce the risk of hospitalization for HF by 18–50% in the pooled analysis. Therefore, it is possible that some of the rate reduction we estimated for MACE is driven by the inclusion of HF hospitalization in our definition.

We also found that SGLT-2Is were associated with a 58% reduced rate of HF hospitalization, compared to DPP-4Is. This result was similar in magnitude to Singaporean, Israeli, and Canadian estimates in the CVD-REAL2 study (Kosiborod et al., 2018). When we included the interaction term in our model we estimated a 73% reduction in HF hospitalization rates in people receiving SGLT-2Is versus DPP-4Is. This provides evidence of frailty potentially modifying the effects of SGLT-2Is on HF hospitalization outcomes. In contrast, conditions that are highly prevalent in frail populations such as prior HF, existing CVD and renal impairment have not been shown to modify the effect of SGLT-2Is on HF hospitalizations (Giorgino et al., 2020). Older age, which is strongly associated with frailty, also is not known to alter the beneficial effects of SGLT-2Is on HF outcomes (Abdelhafiz and Sinclair, 2020). At the time of this study, SGLT-2Is were relatively new to the Australian market and the beneficial cardiovascular outcomes demonstrated by the Cardiovascular Outcome Trials (CVOTs) were not yet known (Zinman et al., 2015; Clegg et al., 2018; Perkovic et al., 2018), therefore prescribers may have been more hesitant to prescribe this class of medications to individuals with HF. Our results confirm that HF was less common amongst SGLT-2I (3.0%) compared to DPP-4I (7.3%) recipients. This difference was accounted for using IPTWs, which balanced the baseline clinical characteristics of the exposure and comparator groups, thus minimizing the effects of prescriber bias.

We estimated that SGLT-2Is are associated with a 62% reduction in the rate of mortality compared to DPP-4Is among our hospitalized cohort. This result was similar to a United Kingdom study of The Health Improvement Network (THIN) database (Toulis et al., 2017), which estimated that dapagliflozin was associated with half the rate of all-cause death, compared to other antihyperglycemic treatments (Toulis et al., 2017). It was also similar to the mortality estimates from the CVD-REAL2 (Kohsaka et al., 2020). Suissa et al. suggest that some all-cause mortality estimates such as those in CVD-REAL2 may be exaggerated by immortal time bias, resulting from a longer duration of DPP-4I use and possibly a more extended history of type 2 diabetes compared to SGLT-2Is (Suissa, 2018). Therefore, we included prior use of DPP-4Is as a baseline covariate in our multivariate model to account for those who had taken this medication class in the past. Finally, Australian and international guidelines caution against the intensification of type 2 diabetes regimens for frail individuals and those with important comorbidities or limited life expectancy (The Royal Australian College of General Practitioners (RACGP), 2016; Garber et al., 2020; American Diabetes Association, 2019; Cornell, 2017). It was not possible to ascertain clinicians’ perception of poor prognosis from our dataset, but this may constitute an unmeasured confounder that explains the lower incidence of mortality amongst those prescribed SGLT-2Is.

### Strengths and Limitations

This was the first study to examine cardiovascular outcomes and all-cause mortality associated with SGLT-2Is in people who are frail and non-frail. We analyzed data from all Victorian public and private hospitals over four and a half years. Data were available on all reimbursed prescriptions dispensed through community pharmacies and at hospital discharge. Confounding by disease severity was minimized because SGLT-2Is and DPP-4Is are both second-line agents. We used a treatment decision design (Brookhart, 2015) rather than an incident user design, and it was possible that patients used SGLT-2Is or DPP-4Is before their index discharge, which might have resulted in a bias related to the differential start dates of SGLT-2Is or DPP-4Is. To account for this, we adjusted out model for prior use of DPP-4Is. The treatment decision design is relevant to clinical practice because hospital discharge represents a time when clinicians decide to initiate, continue, or discontinue treatment, however results may not be generalizable to individuals who have not been recently hospitalized. Moreover, we could not be sure that individuals identified as being SGLT-2I users during the landmark period did not switch to DPP-4Is during follow-up and vice versa, nor could we determine exact medication initiation dates. The HFRS was originally validated in people aged >75 years and our study population contained patients aged ≥30 years. Data were not available on each patient’s glycated hemoglobin or lifestyle. Duration of type 2 diabetes was also unknown, although the DCSI scores acted as surrogates for duration. Finally, we analyzed medication dispensing data and it was not possible to determine if patients dispensed SGLT-2I or DPP-4Is took these medications as prescribed and dispensed.

## Conclusion

Our results suggest SGLT-2Is have clear advantages over DPP-4Is with respect to rates of MACE, HF hospitalizations and all-cause mortality in both frail and non-frail people. Our study provides preliminary evidence to suggest that SGLT-2Is may be preferred to DPP-4Is in the treatment of frail people living with type 2 diabetes, which could inform the development of updated type 2 diabetes clinical practice guidelines.

## Data Availability

The individual level data supporting the conclusions of this article are only available to approved researchers, however the authors will make all reasonable attempts to share aggregate level data, upon request.

## Abbreviations

AIHW, Australian Institute of Health and Welfare; ATC, Anatomical Therapeutic Chemical; CABG, Coronary Artery Bypass Graft; CI, Confidence interval; CVOT, Cardiovascular Outcome Trial; DCSI, Diabetes Complications Severity Index; DPP-4I, Dipeptidyl peptidase-4 inhibitors; EMPA-REG OUTCOMES, The Empagliflozin, Cardiovascular Outcomes, and Mortality in type 2 Diabetes trial; HF, Heart failure; HFRS, Hospital Frailty Risk Score; HR, Hazard Ratio; ICD, International Classification of Diseases; IPTW, Inverse Probability of Treatment Weight; MACE, Major adverse cardiovascular event; MI, Myocardial Infarction; PBS, Pharmaceutical Benefits Scheme; PCI, Percutaneous Coronary Interventions; RCT, Randomized controlled trials; SAVOR-TIMI-53, The Saxagliptin Assessment of Vascular Outcomes Recorded in Patients with Diabetes Mellitus–Thrombolysis in Myocardial Infarction 53 trial; SGLT-2I, Sodium-glucose cotransporter-2 inhibitors; sHR, Subdistribution Hazard ratio; SMD, Standardized Mean Difference; THIN, The Health Improvement Network; VAED, Victorian Admitted Episodes Dataset.

## References

[B1] Abdelhafiz A. H. McNicholas E. Sinclair A. J. (2016). Hypoglycemia, Frailty and Dementia in Older People with Diabetes: Reciprocal Relations and Clinical Implications. J. Diabetes Complicat. 30 (8), 1548–1554. 10.1016/j.jdiacomp.2016.07.027 27524280

[B2] Abdelhafiz A. H. Sinclair A. J. (2020). Cardio-renal Protection in Older People with Diabetes with Frailty and Medical Comorbidities - A Focus on the New Hypoglycaemic Therapy. J. Diabetes Complicat. 34 (9), 107639. 10.1016/j.jdiacomp.2020.107639 32595017

[B3] American Diabetes Association (2019). Pharmacologic Approaches to Glycemic Treatment: Standards of Medical Care in Diabetes. Diabetes Care 42 (Suppl. 1), S90. 10.2337/dc19-S009 30559235

[B4] Austin P. C. (2009). Balance Diagnostics for Comparing the Distribution of Baseline Covariates between Treatment Groups in Propensity-Score Matched Samples. Stat. Med. 28 (25), 3083–3107. 10.1002/sim.3697 19757444 PMC3472075

[B5] Brookhart M. A. (2015). Counterpoint: The Treatment Decision Design. Am. J. Epidemiol. 182 (10), 840–845. 10.1093/aje/kwv214 26507307 PMC4634307

[B6] Chang H. Y. Weiner J. P. Richards T. M. Bleich S. N. Segal J. B. (2012). Validating the Adapted Diabetes Complications Severity Index in Claims Data. Am. J. Manag. Care 18 (11), 721–726. 23198714

[B7] Clegg L. Heerspink H. L. Penland R. C. Tang W. Boulton D. W. Bachina S. (2018). “Impact of SGLT2 Inhibitors (SGLT2i) on Cardiovascular (CV) Risk and Estimated Glomerular Filtration Rate (eGFR) in the EXSCEL Placebo Group,” in Diabetes. Alexandria, VA: LB, 67 Suppl. 1, 130. 10.2337/db18-130-lb

[B8] Cornell S. (2017). Comparison of the Diabetes Guidelines from the ADA/EASD and the AACE/ACE. J. Am. Pharm. Assoc. (2003) 57 (2), 261–265. 10.1016/j.japh.2016.11.005 28065547

[B9] Dugan J. Shubrook J. (2017). International Classification of Diseases, 10th Revision, Coding for Diabetes. Clin. Diabetes 35 (4), 232–238. 10.2337/cd16-0052 29109613 PMC5669129

[B10] Fei Y. Tsoi M. F. Cheung B. M. Y. (2019). Cardiovascular Outcomes in Trials of New Antidiabetic Drug Classes: a Network Meta-Analysis. Cardiovasc Diabetol. 18 (1), 112. 10.1186/s12933-019-0916-z 31462224 PMC6714383

[B11] Filion K. B. Lix L. M. Yu O. H. Dell'Aniello S. Douros A. Shah B. R. (2020). Sodium Glucose Cotransporter 2 Inhibitors and Risk of Major Adverse Cardiovascular Events: Multi-Database Retrospective Cohort Study. BMJ 370, m3342. 10.1136/bmj.m3342 32967856 PMC8009082

[B12] Garber A. J. Handelsman Y. Grunberger G. Einhorn D. Abrahamson M. J. Barzilay J. I. (2020). Consensus Statement by the American Association of Clinical Endocrinologists and American College of Endocrinology on the Comprehensive Type 2 Diabetes Management Algorithm. Endocr. Pract. 26 (1), 107–139. 10.4158/CS-2019-0472 32022600

[B13] Gilbert T. Neuburger J. Kraindler J. Keeble E. Smith P. Ariti C. (2018). Development and Validation of a Hospital Frailty Risk Score Focusing on Older People in Acute Care Settings Using Electronic Hospital Records: an Observational Study. Lancet 391 (10132), 1775–1782. 10.1016/S0140-6736(18)30668-8 29706364 PMC5946808

[B14] Giorgino F. Vora J. Fenici P. Solini A. (2020). Cardiovascular Protection with Sodium-Glucose Co-transporter-2 Inhibitors in Type 2 Diabetes: Does it Apply to All Patients? Diabetes Obes. Metab. 22 (9), 1481–1495. 10.1111/dom.14055 32285611 PMC7496739

[B15] Glasheen W. P. Renda A. Dong Y. (2017). Diabetes Complications Severity Index (DCSI)-Update and ICD-10 Translation. J. Diabetes Complicat. 31 (6), 1007–1013. 10.1016/j.jdiacomp.2017.02.018 28416120

[B16] Ibrahim M. Baker J. Cahn A. Eckel R. H. El Sayed N. A. Fischl A. H. (2020). Hypoglycaemia and its Management in Primary Care Setting. Diabetes Metab. Res. Rev. 36 (8), e3332. 10.1002/dmrr.3332 32343474

[B17] Ida S. Kaneko R. Imataka K. Murata K. (2019). Relationship between Frailty and Mortality, Hospitalization, and Cardiovascular Diseases in Diabetes: a Systematic Review and Meta-Analysis. Cardiovasc Diabetol. 18 (1), 81. 10.1186/s12933-019-0885-2 31215496 PMC6582520

[B18] Kaneko M. Narukawa M. (2016). Meta-analysis of Dipeptidyl Peptidase-4 Inhibitors Use and Cardiovascular Risk in Patients with Type 2 Diabetes Mellitus. Diabetes Res. Clin. Pract. 116, 171–182. 10.1016/j.diabres.2016.04.012 27321333

[B19] Karagiannis T. Bekiari E. Boura P. Tsapas A. (2016). Cardiovascular Risk with DPP-4 Inhibitors: Latest Evidence and Clinical Implications. Ther. Adv. Drug Saf. 7 (2), 36–38. 10.1177/2042098615623915 27034771 PMC4785858

[B20] Kohsaka S. Lam C. S. P. Kim D. J. Cavender M. A. Norhammar A. Jørgensen M. E. (2020). Risk of Cardiovascular Events and Death Associated with Initiation of SGLT2 Inhibitors Compared with DPP-4 Inhibitors: an Analysis from the CVD-REAL 2 Multinational Cohort Study. Lancet Diabetes Endocrinol. 8 (7), 606–615. 10.1016/S2213-8587(20)30130-3 32559476

[B21] Kosiborod M. Lam C. S. P. Kohsaka S. Kim D. J. Karasik A. Shaw J. (2018). Cardiovascular Events Associated with SGLT-2 Inhibitors versus Other Glucose-Lowering Drugs: The CVD-REAL 2 Study. J. Am. Coll. Cardiol. 71 (23), 2628–2639. 10.1016/j.jacc.2018.03.009 29540325

[B22] Mi X. Hammill B. G. Curtis L. H. Lai E. C. Setoguchi S. (2016). Use of the Landmark Method to Address Immortal Person-Time Bias in Comparative Effectiveness Research: a Simulation Study. Stat. Med. 35 (26), 4824–4836. 10.1002/sim.7019 27350312

[B23] National Institute for Health and Care Excellence (NICE) (2017). *Type 2 Diabetes in Adults: Management’*, *NICE Guideline NG28* . Internet. London, United Kingdom: National Institute for Health and Care Excellence. [cited 2021 Nov. 22]. Available from: https://www.nice.org.uk/guidance/ng28.

[B24] Onder G. Vetrano D. L. Marengoni A. Bell J. S. Johnell K. Palmer K. (2018). Accounting for Frailty when Treating Chronic Diseases. Eur. J. Intern Med. 56, 49–52. 10.1016/j.ejim.2018.02.021 29526651

[B25] Perkisas S. Vandewoude M. (2016). Where Frailty Meets Diabetes. Diabetes Metab. Res. Rev. 32 (S1), 261–267. 10.1002/dmrr.2743 26453435

[B26] Perkovic V. de Zeeuw D. Mahaffey K. W. Fulcher G. Erondu N. Shaw W. (2018). Canagliflozin and Renal Outcomes in Type 2 Diabetes: Results from the CANVAS Program Randomised Clinical Trials. LancetDiabetes Endocrinol. 6 (9), 691–704. 10.1016/S2213-8587(18)30141-4 29937267

[B27] Scirica B. M. Bhatt D. L. Braunwald E. Steg P. G. Davidson J. Hirshberg B. (2013). Saxagliptin and Cardiovascular Outcomes in Patients with Type 2 Diabetes Mellitus. N. Engl. J. Med. 369 (14), 1317–1326. 10.1056/NEJMoa1307684 23992601

[B28] State Government of Victoria (2019). Victorian Admitted Episodes Dataset Manual 2019-2020. Internet. Melbourne: Department of Health & Human Services. [cited 2021 Nov. 22]. Available from: https://www.health.vic.gov.au/data-reporting/victorian-admitted-episodes-dataset.

[B29] Suissa S. (2018). Lower Risk of Death with SGLT2 Inhibitors in Observational Studies: Real or Bias? Diabetes Care 41 (1), e109. 10.2337/dci18-0015 29263192

[B30] The Royal Australian College of General Practitioners (RACGP) (2016). Management Of Type 2 Diabetes: A Handbook for General Practice 2016-2018. Internet. Melbourne: The Royal Australian College of General Practitioners. [cited 2021 Nov. 22]. Available from: https://www.racgp.org.au/getattachment/41fee8dc-7f97-4f87-9d90-b7af337af778/Management-of-type-2-diabetes-A-handbook-for-general-practice.aspx.

[B31] Thoemmes F. Ong A. D. (2015). A Primer on Inverse Probability of Treatment Weighting and Marginal Structural Models. Emerg. Adulthood 4 (1), 40–59. 10.1177/2167696815621645

[B32] Toulis K. A. Willis B. H. Marshall T. Kumarendran B. Gokhale K. Ghosh S. (2017). All-Cause Mortality in Patients with Diabetes under Treatment with Dapagliflozin: A Population-Based, Open-Cohort Study in the Health Improvement Network Database. J. Clin. Endocrinol. Metab. 102 (5), 1719–1725. 10.1210/jc.2016-3446 28323967

[B33] Wiviott S. D. Raz I. Sabatine M. S. (2018). Dapagliflozin and Cardiovascular Outcomes in Type 2 Diabetes. N. Engl. J. Med. 380 (4), 1881–1882. 10.1056/NEJMc1902837 31067395

[B34] Wood S. Bell J. S. Magliano D. J. Fanning L. Cesari M. Keen C. S. (2021). Impact of Age, Frailty, and Dementia on Prescribing for Type 2 Diabetes at Hospital Discharge 2012-2016. J. Frailty Aging 10 (4), 343–349. 10.14283/jfa.2021.6 34549249

[B35] Zinman B. Wanner C. Lachin J. M. Fitchett D. Bluhmki E. Hantel S. (2015). Empagliflozin, Cardiovascular Outcomes, and Mortality in Type 2 Diabetes. N. Engl. J. Med. 373 (22), 2117–2128. 10.1056/NEJMoa1504720 26378978

