# Supplementary material for: Effectiveness of Sodium-Glucose Cotransporter-2 Inhibitors vs. Dipeptidyl Peptidase-4 Inhibitors in Frail People With Diabetes Who Were Recently Hospitalized
Source: Front Pharmacol. 2022 Jul 12;13:886834. doi: 10.3389/fphar.2022.886834 (PMC9315378; doi:10.3389/fphar.2022.886834)
Supplement: Supplementary file 1 [file DataSheet1.docx]

### Appendix A: Anatomical Therapeutic Chemical (ATC) codes to identify medications

| **Drug class** | **ATC code** |
| --- | --- |
| **Exposure medications**  SGLT-2Is | A10BK, A10BX (individual), A10BD15-16, A10BD19-21, A10BD23-25 (in combination) |
| DPP-4Is | A10BH (individual), A10BD10-13, A10BD18-19, A10BD21-22, A10BD24-25 (in combination) |
| **Baseline medications***  Statin | C10AA, C10BA, C10BX |
| ACEI/ARB | C09A, C09B, C09C, C09D (exclude C09DX04) |
| Anti-dementia drugs | N06D |
| Beta-blockers | C07 |
| Antiplatelets  Insulins | B01AC  A10A |
| Other lipid-lowering medications | C10AB, C10AC, C10AD, C10AX |
| Glucose lowering medications | A10A and A10B |
| Anticoagulant | B01AA |
| Antipsychotic | N05A |
| Aldosterone Antagonists | C03DA |
| Digoxin | C01AA05 |
| Calcium channel blockers | C08C |
| Loop diuretics and thiazides | C03C (loop diuretics), C03A (thiazides), |

SGLT-2I Sodium Glucose Cotransporter-2 Inhibitor; DPP-4I Dipeptidyl Peptidase-4 Inhibitor; ACE Angiotensin Converting Enzyme Inhibitor; ARB Angiotensin 2 Receptor Blocker

* Baseline medications are identified using a fixed one-year period prior to index hospitalization.

### Appendix B: Specification of relevant prior medical conditions

| **Medical condition** | **ICD-10-AM** | **Time frame** |
| --- | --- | --- |
| Angina | I20.0, I20.1, I20.8, I20.9 | 8 years prior to index date |
| MI | I21-23 | 8 years prior to index date |
| Hypertension | I10-15 | 8 years prior to index date |
| Heart failure | I50 | 8 years prior to index date |
| Atrial fibrillation | I48 | 8 years prior to index date |
| Stroke | I60-I64 | 8 years prior to index date |
| Peripheral vascular disease | I70-I73 | 8 years prior to index date |
| Chronic kidney disease | E10.2, E11.2, E12.2, E13.2, E14.2, N00-02, N04-08, N11-12, N14-16, N25-28, T82.4, T86.1, Q60- 63, Z94.0, Z99.2, Z49, N17-19 | 8 years prior to index date |
| Dementia | F01-F03, G30, U79.1 | 8 years prior to index date |
|  |  |  |
| Dialysis | Z49 | 8 years prior to index date |
| Diabetic mono-/polyneuropathy | G99.0, G59.0, G63.2, E10.4, E11.4, E12.4, E13.4, E14.4 | 8 years prior to index date |
| Diabetic eye disease | H28.0, H35.8, H36.0, E10.3, E11.3, E12.3, E13.3, E14.3 | 8 years prior to index date |
| Diabetic foot/Peripheral angiopathy | E11.6B, M14.2, M14.6, M90.8, L98.4, E10.5, E11.5, E12.5, E13.5, E14.5 | 8 years prior to index date |
| Other diabetic complications | E11.6, E10.6, E13.6, E14.6, E10.7, E11.7, E12.7, E13.7, E14.7, E10.8, E11.8, E12.0, E12.8, E13.8, E14.8 | 8 years prior to index date |
| Severe hypoglycemia | E10.0, E11.0, E12.0, E13.0, E14.0, E11.6A, E16.0-2 | <I year prior to index date |
| Keto-/lactate acidosis | E10.1, E11.1, E12.1, E13.1, E14.1, E87.2 | <I year prior to index date |
| Lower limb amputations | Z89 | <I year prior to index date |
| Cancer | C00-C99 | <I year prior to index date |
| COPD | J44 | 8 years prior to index date |
| Frailty index* |  | Calculated on index hospitalization |

*Gilbert et al. Lancet 391:1775-1782, 2018

ICD-10-AM International Classification of Diseases (Australian Modified) codes, 10^th^ edition; CVD Cardiovascular disease; COPD Chronic Obstructive Pulmonary Disease

### Appendix C: Definition of MACE events

| **Event** | **Definition** |
| --- | --- |
| MI | Hospitalization with a principal diagnosis of MI (ICD10: I21-23) |
| CABG | Hospitalisation with a procedure code for: 3530400, 3530401, 3850500, 3530500, 3530501, 3531005, 9020100, 3845619, 3865308, 3849700, 3849701, 3849702, 3850003, 3849704, 3849705, 3849706, 3849707, 3850002, 3850004, 3850302, 3850303, 3850304, 3863700, 3850000, 3850001, 3850300, 3850301, 9022100, 9020101, 9020102, 9020103 |
| PCI with Stent | Hospitalisation with a procedure code for: 353100, 3531001, 3531002, 3830600, 3830601, 3830602, 3533800, 3534401, 3831200, 3831201, 3831800, 3831801 |
| Heart Failure | Hospitalization with a principal diagnosis of heart failure (ICD10: I50) |
| Stroke | Hospitalization with a principal diagnosis of stroke (ICD10: I60-I64) |
| Revascularization | Hospitalization with a principal diagnosis of revascularization (ICD10: Z95) |
| CVD death* | Death with any of the above as a primary cause of death |

*If a patient died during the follow-up and the primary cause of death was indicated by any of the above ICD-10 codes, then an event was recorded. If the cause of death was not one of the above then a competing risk event was recorded. If no event or competing risk occurred during follow-up then patients were censored on June 30^th^, 2018.

**Appendix D:** List of 109 ICD-10 codes and number of points awarded for each to create the hospital frailty risk score (HFRS) [21].

| **ICD Code and Description** | **HFRS value** |
| --- | --- |
| F00 Dementia in Alzheimer's disease | 7.1 |
| G81 Hemiplegia | 4.4 |
| G30 Alzheimer's disease | 4.0 |
| I69 Sequelae of cerebrovascular disease (secondary codes) | 3.7 |
| R29 Other symptoms and signs involving the nervous and musculoskeletal systems (R29·6 Tendency to fall) | 3.6 |
| N39 Other disorders of urinary system (includes urinary tract infection and urinary incontinence) | 3.2 |
| F05 Delirium, not induced by alcohol and other psychoactive substances | 3.2 |
| W19 Unspecified fall | 3.2 |
| S00 Superficial injury of head | 3.2 |
| R31 Unspecified haematuria | 3.0 |
| B96 Other bacterial agents as the cause of diseases classified to other chapters (secondary code) | 2.9 |
| R41 Other symptoms and signs involving cognitive functions and awareness | 2.7 |
| R26 Abnormalities of gait and mobility | 2.6 |
| I67 Other cerebrovascular diseases | 2.6 |
| R56 Convulsions, not elsewhere classified | 2.6 |
| R40 Somnolence, stupor and coma | 2.5 |
| T83 Complications of genitourinary prosthetic devices, implants and grafts | 2.4 |
| S06 Intracranial injury | 2.4 |
| S42 Fracture of shoulder and upper arm | 2.3 |
| E87 Other disorders of fluid, electrolyte and acidbase balance | 2.3 |
| M25 Other joint disorders, not elsewhere classified | 2.3 |
| E86 Volume depletion | 2.3 |
| R54 Senility | 2.2 |
| Z50 Care involving use of rehabilitation procedures | 2.1 |
| F03 Unspecified dementia | 2.1 |
| W18 Other fall on same level | 2.1 |
| Z75 Problems related to medical facilities and other health care | 2.0 |
| F01 Vascular dementia | 2.0 |
| S80 Superficial injury of lower leg | 2.0 |
| L03 Cellulitis | 2.0 |
| H54 Blindness and low vision | 1.9 |
| E53 Deficiency of other B group vitamins | 1.9 |
| Z60 Problems related to social environment | 1.8 |
| G20 Parkinson's disease | 1.8 |
| R55 Syncope and collapse | 1.8 |
| S22 Fracture of rib(s), sternum and thoracic spine | 1.8 |
| K59 Other functional intestinal disorders | 1.8 |
| N17 Acute renal failure | 1.8 |
| L89 Decubitus ulcer | 1.7 |
| Z22 Carrier of infectious disease | 1.7 |
| B95 Streptococcus and staphylococcus as the cause of diseases classified to other chapters | 1.7 |
| L97 Ulcer of lower limb, not elsewhere classified | 1.6 |
| R44 Other symptoms and signs involving general sensations and perceptions | 1.6 |
| K26 Duodenal ulcer | 1.6 |
| I95 Hypotension | 1.6 |
| N19 Unspecified renal failure | 1.6 |
| A41 Other septicaemia | 1.6 |
| Z87 Personal history of other diseases and conditions | 1.5 |
| J96 Respiratory failure, not elsewhere classified | 1.5 |
| X59 Exposure to unspecified factor | 1.5 |
| M19 Other arthrosis | 1.5 |
| G40 Epilepsy | 1.5 |
| M81 Osteoporosis without pathological fracture | 1.4 |
| S72 Fracture of femur | 1.4 |
| S32 Fracture of lumbar spine and pelvis | 1.4 |
| E16 Other disorders of pancreatic internal secretion | 1.4 |
| R94 Abnormal results of function studies | 1.4 |
| N18 Chronic renal failure | 1.4 |
| R33 Retention of urine | 1.3 |
| R69 Unknown and unspecified causes of morbidity | 1.3 |
| N28 Other disorders of kidney and ureter, not elsewhere classified | 1.3 |
| R32 Unspecified urinary incontinence | 1.2 |
| G31 Other degenerative diseases of nervous system, not elsewhere classified | 1.2 |
| Y95 Nosocomial condition | 1.2 |
| S09 Other and unspecified injuries of head | 1.2 |
| R45 Symptoms and signs involving emotional state | 1.2 |
| G45 Transient cerebral ischaemic attacks and related syndromes | 1.2 |
| Z74 Problems related to care-provider dependency | 1.1 |
| M79 Other soft tissue disorders, not elsewhere classified | 1.1 |
| W06 Fall involving bed | 1.1 |
| S01 Open wound of head | 1.1 |
| A04 Other bacterial intestinal infections | 1.1 |
| A09 Diarrhoea and gastroenteritis of presumed infectious origin | 1.1 |
| J18 Pneumonia, organism unspecified | 1.1 |
| J69 Pneumonitis due to solids and liquids | 1.0 |
| R47 Speech disturbances, not elsewhere classified | 1.0 |
| E55 Vitamin D deficiency | 1.0 |
| Z93 Artificial opening status | 1.0 |
| R02 Gangrene, not elsewhere classified | 1.0 |
| R63 Symptoms and signs concerning food and fluid intake | 0.9 |
| H91 Other hearing loss | 0.9 |
| W10 Fall on and from stairs and steps | 0.9 |
| W01 Fall on same level from slipping, tripping and stumbling | 0.9 |
| E05 Thyrotoxicosis [hyperthyroidism] | 0.9 |
| M41 Scoliosis | 0.9 |
| R13 Dysphagia | 0.8 |
| Z99 Dependence on enabling machines and devices | 0.8 |
| U80 Agent resistant to penicillin and related antibiotics | 0.8 |
| M80 Osteoporosis with pathological fracture | 0.8 |
| K92 Other diseases of digestive system | 0.8 |
| I63 Cerebral Infarction | 0.8 |
| N20 Calculus of kidney and ureter | 0.7 |
| F10 Mental and behavioural disorders due to use of alcohol | 0.7 |
| Y84 Other medical procedures as the cause of abnormal reaction of the patient | 0.7 |
| R00 Abnormalities of heart beat | 0.7 |
| J22 Unspecified acute lower respiratory infection | 0.7 |
| Z73 Problems related to life-management difficulty | 0.6 |
| R79 Other abnormal findings of blood chemistry | 0.6 |
| Z91 Personal history of risk-factors, not elsewhere classified | 0.5 |
| S51 Open wound of forearm | 0.5 |
| F32 Depressive episode | 0.5 |
| M48 Spinal stenosis (secondary code only) | 0.5 |
| E83 Disorders of mineral metabolism | 0.4 |
| M15 Polyarthrosis | 0.4 |
| D64 Other anaemias | 0.4 |
| L08 Other local infections of skin and subcutaneous tissue | 0.4 |
| R11 Nausea and vomiting | 0.3 |
| K52 Other noninfective gastroenteritis and colitis | 0.3 |
| R50 Fever of unknown origin | 0.1 |

**Appendix E:** Diabetes Complications and Severity Index (DCSI) scores assigned with relevant ICD-10 codes [22].

| **DCSI score** | **ICD-10 Codes** | **Description of Codes** |
| --- | --- | --- |
| **Ophthalmic** | | |
| 1 | Main Codes  E08    E09    E10  E11  E13  Relevant Subcodes  E**.3x, excluding  E**.34x  & E**.35x | Diabetes Mellitus due to underlying conditions  Drug or chemical induced diabetes mellitus  Type 1 diabetes mellitus  Type 2 diabetes mellitus  Other specified diabetes mellitus  With ophthalmic complications |
| 1 | H35.0x | Background retinopathy and retinal vascular changes |
| 1 | H35.35x | Cystoid macular degeneration |
| 1 | H35.6x  H35.8x  H35.9 | Retinal hemorrhage  Other specified retinal disorders  Unspecified retinal disorder |
| 2 | H33.x | Retinal detachments and breaks |
| 2 | E**.34x | Severe nonproliferative diabetic retinopathy  Proliferative diabetic retinopathy |
| 2 | H54.x | Blindness and low vision |
| 2 | H43.1x | Vitreous hemorrhage |
| **Nephropathy** | | |
| 1 | Main Codes  E08  E09  E10  E11  E13  Relevant Subcodes  E**.21  E**.22  E**.29 | Diabetes mellitus due to underlying condition  Drug or chemical induced diabetes mellitus  Type 1 diabetes mellitus  Type 2 diabetes mellitus  Other specified diabetes mellitus  With diabetic nephropathy  With diabetic chronic kidney disease  With other diabetic kidney complication |
| 1 | N00.x | Acute nephritic syndrome |
| 1 | N04.x | Nephrotic syndrome |
| 1 | N03.x | Chronic nephritic syndrome |
| 1 | N05.x | Unspecified nephritic syndrome |
| 1 | N18.1 | CKD, Stage 1 |
| 1 | N18.2 | CKD, Stage 2 (mild) |
| 1 | N18.3 | CKD, Stage 3 (moderate) |
| 1 | N18.9 | CKD, unspecified |
| 2 | N18.4 | CKD, Stage 4 (severe) |
| 2 | N18.5 | CKD, Stage 5 |
| 2 | N18.6 | End stage renal disease |
| 2 | N19 | Unspecified kidney failure |
| **Neuropathy** | | |
| 1 | Main Codes  E08  E09  E10  E11  E13  Relevant Subcodes  E**.4x | Diabetes mellitus due to underlying condition  Drug or chemical induced diabetes mellitus  Type 1 diabetes mellitus  Type 2 diabetes mellitus  Other specified diabetes mellitus  With neurological complications |
| 1 | G90.09 | Other [than carotid sinus syncope] idiopathic peripheral autonomic neuropathy |
| 1 | G90.8; G90.9; G99.0 | Other disorders of autonomic nervous system; Disorder of the autonomic nervous system, unspecified; Autonomic neuropathy in diseases classified elsewhere |
| 1 | G56.x | Mononeuropathies of upper limb |
| 1 | G57.x | Mononeuropathies of lower limb |
| 1 | G60.9 | Hereditary and idiopathic neuropathy, unspecified |
| 1 | G73.3 | Myasthenic syndromes in other diseases classified elsewhere |
| 1 | G90.01 | Carotid sinus syncope |
| 1 | H49.x | Paralytic strabismus |
| 1 | I95.1 | Orthostatic hypotension |
| 1 | K31.84 | Gastroparesis |
| 1 | K59.1 | Functional diarrhea |
| 1 | N31.9 | Neuromuscular dysfunction of bladder, unspecified |
| 1 | M14.6x | Charcôt’s joint |
| 1 | S04.x | Injury to cranial nerve |
| **Cerebrovascular** | | |
| 1 | G45.x | Transient cerebral ischemic attacks and related syndromes |
| 2 | I61.x | Nontraumatic intracerebral hemorrhage |
| 2 | I63.x | Cerebral infarction |
| 2 | I65.x | Occlusion and stenosis of precerebral arteries, not resulting in cerebral infarction |
| 2 | I66.x | Occlusion and stenosis of cerebral arteries, not resulting in cerebral infarction |
| 2 | I67.81 | Acute cerebrovascular insufficiency |
| **Cardiovascular** | | |
| 1 | I24.x | Other acute IHD |
| 1 | I20.x | Angina pectoris |
| 1 | I25.x, excluding I25.2 | Chronic ischemic heart disease |
| 1 | I70.x, excluding I70.25 & I70.26x | Atherosclerosis |
| 2 | I21.x | STEMI and NSTEMI |
| 2 | I22.x | Subsequent STEMI and NSTEMI |
| 2 | I23.x | Complications following STEMI and NSTEMI |
| 2 | I25.2 | Old myocardial infarction |
| 2 | I48.x | Atrial fibrillation and flutter |
| 2 | I46.x | Cardiac arrest |
| 2 | I47.x | Paroxysmal tachycardia |
| 2 | I49.x | Other cardiac arrhythmias |
| 2 | I50.x | Heart failure |
| 2 | I70.25/170.26x | Atherosclerosis of native arteries of the extremities with ulceration/gangrene |
| 2 | I71.x | Aortic aneurysm/dissection |
| **Peripheral Vascular Disease** | | |
| 1 | Main Codes  E08  E09  E10  E11  E13  Relevant Subcodes  E**.51  E**.59  E**.621 | Diabetes mellitus due to underlying condition  Drug or chemical induced diabetes mellitus  Type 1 diabetes mellitus  Type 2 diabetes mellitus  Other specified diabetes mellitus  Diabetic peripheral angiopathy, no gangrene  Diabetes, other circulatory complications  Diabetic foot ulcer |
| 1 | I72.4 | Aneurysm of artery of lower extremity |
| 1 | I70.21x, I73.89,  I73.9 | Atherosclerosis of native arteries of extremities with intermittent claudication, Other specified peripheral vascular diseases, Peripheral vascular disease, unspecified |
| 1 | S91.3x | Open wound of foot |
| 2 | A48.0 | Gas gangrene |
| 2 | I74.3 | Embolism and thrombosis of arteries of the lower extremities |
| 2 | L97.x | Non-pressure chronic ulcer of lower limb, not elsewhere classified |
| 2 | E**.52  I96 | Diabetic peripheral angiopathy, with gangrene  Gangrene, not elsewhere classified |
| **Metabolic** | | |
| 1 | Main Codes  E08  E09  E10  E11  E13  Relevant Subcodes  E**.00  E**.10  E**.649 | Diabetes mellitus due to underlying condition  Drug or chemical induced diabetes mellitus  Type 1 diabetes mellitus  Type 2 diabetes mellitus  Other specified diabetes mellitus  With hyperosmolarity, without nonketotic hyperglycemic-hyperosmolar coma (NKHHC)  With ketoacidosis, without coma  With hypoglycemia, without coma |
| 2 | Main Codes  E08  E09  E10  E11  E13  Relevant Subcodes  E**.01  E**.11  E**.641 | Diabetes mellitus due to underlying condition  Drug or chemical induced diabetes mellitus  Type 1 diabetes mellitus  Type 2 diabetes mellitus  Other specified diabetes mellitus  With hyperosmolarity, with coma  With ketoacidosis, with coma  With hypoglycemia, with coma |

*The character ‘x’ to the right of a decimal point indicates that 1 or more digits must be added to the main 3 digits to create a billable code.

**Appendix F:** Proportions of people with and without a history of DPP-4I dispensings in the one year prior to index date allocated to the DPP-4I or SGLT-2I cohorts.

| **Total (N=32,043)** | **Dispensed DPP-4I in the year prior to index date (N=25,727)** | **Not dispensed DPP-4I in the year prior to index date (N=6,316)** |
| --- | --- | --- |
| **Allocated to DPP-4I cohort** | 24,909 (96.8) | 1,982 (31.4) |
| **Allocated to SGLT-2I cohort** | 818 (3.2) | 4,334 (68.6) |
